# A Bayesian Approach for Modeling Cattle Movements in the United States: Scaling up a Partially Observed Network

**DOI:** 10.1371/journal.pone.0053432

**Published:** 2013-01-04

**Authors:** Tom Lindström, Daniel A. Grear, Michael Buhnerkempe, Colleen T. Webb, Ryan S. Miller, Katie Portacci, Uno Wennergren

**Affiliations:** 1 Department of Physics, Chemistry and Biology, Linköping University, Linköping, Sweden; 2 School of Biological Sciences, University of Sydney, Sydney, New South Wales, Australia; 3 Department of Biology, Colorado State University, Fort Collins, Colorado, United States of America; 4 United States Department of Agriculture, Animal and Plant Health Inspection Service, Center for Epidemiology and Animal Health, Fort Collins, Colorado, United States of America; Universidad de Zarazoga, Spain

## Abstract

Networks are rarely completely observed and prediction of unobserved edges is an important problem, especially in disease spread modeling where networks are used to represent the pattern of contacts. We focus on a partially observed cattle movement network in the U.S. and present a method for scaling up to a full network based on Bayesian inference, with the aim of informing epidemic disease spread models in the United States. The observed network is a 10% state stratified sample of Interstate Certificates of Veterinary Inspection that are required for interstate movement; describing approximately 20,000 movements from 47 of the contiguous states, with origins and destinations aggregated at the county level. We address how to scale up the 10% sample and predict unobserved intrastate movements based on observed movement distances. Edge prediction based on a distance kernel is not straightforward because the probability of movement does not always decline monotonically with distance due to underlying industry infrastructure. Hence, we propose a spatially explicit model where the probability of movement depends on distance, number of premises per county and historical imports of animals. Our model performs well in recapturing overall metrics of the observed network at the node level (U.S. counties), including degree centrality and betweenness; and performs better compared to randomized networks. Kernel generated movement networks also recapture observed global network metrics, including network size, transitivity, reciprocity, and assortativity better than randomized networks. In addition, predicted movements are similar to observed when aggregated at the state level (a broader geographic level relevant for policy) and are concentrated around states where key infrastructures, such as feedlots, are common. We conclude that the method generally performs well in predicting both coarse geographical patterns and network structure and is a promising method to generate full networks that incorporate the uncertainty of sampled and unobserved contacts.

## Introduction

Network analysis is an important technique for extracting epidemiologically relevant information from complex systems. For livestock diseases, animal movement networks have received particular attention because they may serve as a proxy for contact networks for disease spread [1]–[5]). While different diseases have different pathways of transmission, the movement of infected animals between livestock premises is a major risk factor for the introduction of diseases to uninfected herds. Long distance movements are particularly important because they can transmit pathogens great distances from the index herd speeding spread and increasing epidemic size [6]. The use of detailed animal movement data in response to the 2001 Foot and Mouth disease outbreak in the United Kingdom (UK) has spurred considerable advances in the use of contact networks to characterize and predict livestock disease outbreaks in the UK [7], [8], [4]. However, while network models are powerful tools for informing disease spread prediction, data collection may be cumbersome and a complete representation of the network is often impossible to obtain. In situations where the complete network is of interest (e.g. disease spread modeling), some method of scaling up a partially observed network is required. While we focus here on livestock networks, similar problems exist in characterizing wildlife and human contact networks [9]–[10].

In this study we focus on the network of cattle movements in the United States. While considered an important mechanism for disease transmission, the extent of cattle movements in the U.S. is not well characterized, making any surveillance, prediction and control for animal diseases extremely challenging [11]. However, recent work has addressed this deficiency using a sample of Interstate Certificates of Veterinary Inspection (ICVIs), which are required for most non-slaughter movements crossing state lines in the U.S., to develop network models of national cattle shipments (i.e., edges) between counties. The sampling of this network is unique in that we are sampling individual movements that make up weighted edges in the network and do not sample, nor have knowledge about, individual nodes. Also, the sampling is incomplete in two ways. First, observations of movements are based on a 10% sample of ICVIs. Naively scaling up by assuming that each observed edge proportionally represents 10% of actual movements overestimates the number of strong edges (i.e. many sampled movements or strongly weighted edge) and underestimates the connectedness owing to weak edges (i.e. few movements) that are not sampled; both presenting consequences for prediction of outbreak dynamics because we are interested in spatially explicit predictions over the complete network. Second, ICVIs are only required for interstate movements (excluding slaughter), hence movements between counties within states (intrastate) are not reported within this data set. If using the network for epidemiological modeling, the lack of intrastate movements will generate a national network with holes in the structure that will underestimate short distance movements and local disease spread. Finally, modeling cattle movement is not straightforward because the probability of movement is not simply a function of distance. The spatial distribution of infrastructure (e.g., calf producers, feedlots, markets, slaughter facilities) in the U.S. cattle industry creates a source-sink dynamic that also must be addressed.

**Figure 1:**
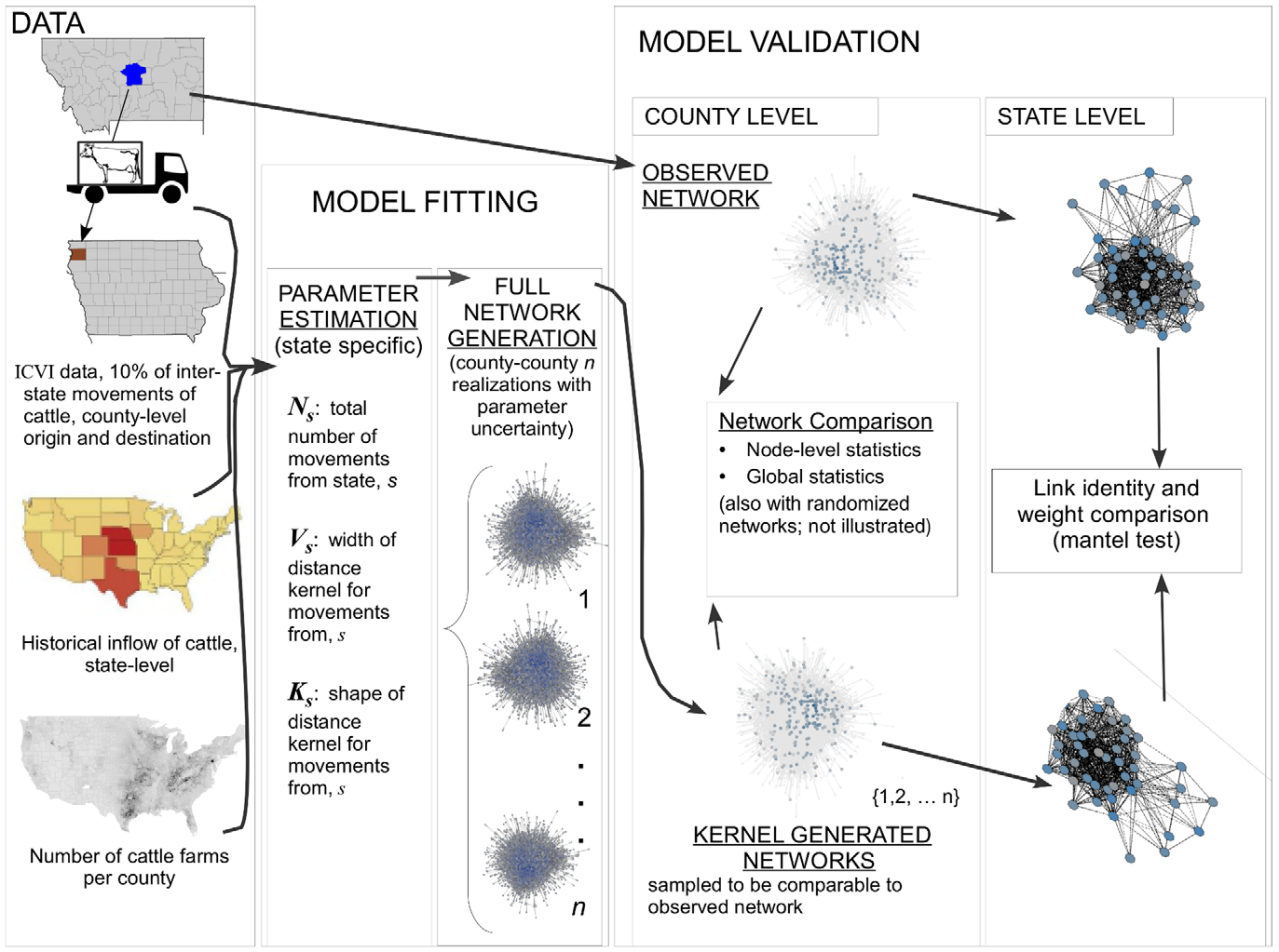
Conceptual diagram of data, model, and validation.

In this paper we present a novel Bayesian kernel approach to address all three issues: (i) 10% sampling, (ii) sampling only interstate movements, and (iii) source-sink dynamics in the U.S. cattle industry. Our aim is to parameterize a spatially explicit probabilistic model for individual movements that may be used for prediction of the whole network structure. Therefore, performance of the model is evaluated by comparing a set of network statistics to the observed network (as given by the ICVI reports) as well as randomized networks. As such, we are fitting the model at a low level (i.e. individual movements) and subsequently evaluating the model performance at a higher level (node-level and global network properties). This paper is structured such that we first introduce the data used for the analysis. We then introduce the kernel and present how parameters are estimated in a Bayesian framework using Markov Chain Monte Carlo (MCMC) simulation. Finally, the model performance is evaluated by comparing networks generated from the posterior predictive distribution of the fitted kernel model with the observed data as well as with randomized networks (Figure 1).

## Materials and Methods

### 2.1 Data

This analysis uses three different data sets. ICVIs provide data on interstate animal movement. Data from the National Agricultural Statistics Service (NASS) describes the current distribution of cattle premises, and a separate NASS survey provides historical measures of cattle flows at the state level.

#### 2.1.1 Interstate Certificate of Veterinary Inspection sampling

ICVIs are an official document required for most interstate cattle movement with the exception of animals going directly to slaughter. In general, ICVIs list the origin and destination addresses for the cattle shipment, number of cattle in the shipment, purpose of shipment, and breed of cattle in the shipment. ICVIs are generally stored as paper documents at the individual states. Characterizing cattle movements requires digitizing a large number of paper documents and sampling is necessary to make data collection feasible. We requested that all states send a 10% sample of their calendar year 2009 cattle ICVIs that originated in their state by taking a systematic sample of every tenth cattle ICVI. We specifically requested origin ICVIs to avoid duplication because copies of ICVIs are maintained by both the sending and receiving states.

We obtained calendar year 2009 ICVIs from 48 states, with the exceptions being New Jersey (did not participate) and Alaska (no ICVIs to report). We excluded Hawaii from the analysis because their contact pattern with other parts of the U.S. is expected to depend on different underlying processes. In general, we successfully obtained a 10% systematic sample of 2009 export ICVIs, but approximations of this sampling design were implemented in Kentucky, Missouri and Vermont to accommodate time and budget constraints.

We created a database of the ICVIs including: origin and destination address; dates the animals were inspected, shipped, and the ICVI was received at the state veterinarian’s office; the purpose of the shipment; whether the shipment was beef or dairy cattle; the number of animals; and the breeds, age, and gender distributions of the cattle in the shipment. In all, this database contains 19,170 interstate shipment records from 2433 counties. We classified shipments as beef or dairy using shipment purpose data on the ICVI. If the production type was not present on the ICVI a classification tree analysis was used to classify the shipment as beef or dairy (Buhnerkempe, unpublished). We aggregated all address information for the origin and destination to the county-level and focus on networks with county as the node and edges as movements between counties, using the county centroids to calculate distances (Figure 1).

#### 2.1.2 Cattle premises

Our model adjusted the probability of movements between counties by the number of premises as reported by the most recent (2007) NASS census of U.S. agriculture. We used data reporting the number of beef and dairy cattle premises per county and define premises as a general term for any type of operation where cattle are traded as a commodity according to the NASS definition: any establishment from which $1,000 or more of agricultural products were sold or would normally be sold during the year (NASS: http://www.nass.usda.gov/About_NASS/History_of_Ag_Statistics/index.asp). We used the 2007 census data to describe the U.S. cattle industry because it is the closest NASS census to the 2009 ICVI data. The census is available for download at http://www.agcensus.usda.gov/Publications/2007/Full_Report/index.asp.

#### 2.1.3 Historical inflow of cattle by state

We also used historical summaries of the number of cattle moved into each U.S. state from other states (inflow) to incorporate national-scale cattle flow patterns. We obtained interstate inflow data from 1988–2009 NASS reports of the total number of cattle imported into each state. The inflows have no information on the states of origin. Historical summaries are available at http://quickstats.nass.usda.gov/.

### 2.2 Kernel Properties and Bayesian Analysis

Here, we describe a novel method based on a Bayesian kernel approach presented in [12], [13]. This approach provides an appropriate way to scale up the 10% sample and allows inference to intrastate movement. It also relates distance information from the ICVI data to source-sink information contained in the NASS census data on number of cattle premises by county and state level historic inflow data. Because the number of cattle premises is reported by county in the NASS census and we aggregate the movement data to county, the model is described at the spatial scale of counties (Figure 1). At the scale of the U.S. there is not comprehensive data available on all types of cattle industry infrastructure within counties. The NASS census reports several types of premises, but excludes important premises types such as markets and slaughter facilities. Therefore, we make the simple assumption that the count of any type of premises is directly related to the probability of interstate movements (section 2.2.1, equation 2).

#### 2.2.1 Model description

We are interested in the joint probability of the total number of movements (*N*, all interstate plus intrastate) and width (*V*) and shape (*K*) parameters of the kernel. This joint distribution is based on data which contains origin ***o***, and destination county, ***d***, of all observed movements as well as location of all counties and the number of premises per county. We want to incorporate parameter uncertainty and rely on Bayesian inference in estimation of parameters *N*, *V* and *K*. The decay in probability of movements with distance is expected to vary between different areas of the U.S. and we therefore estimate different kernel parameters for each state. We assume that the same underlying processes drive interstate and intrastate movements, such that we use the Bayesian inference of the distance dependence to estimate movements regardless of state borders. The likelihood is specified as,
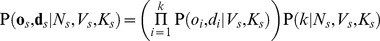
(1)where ***o*** *_s_* and ***d*** *_s_* are the *k* number of observed origin and destination counties for movements from state *s* and *N_s_* is the corresponding (unobserved) total number of movements. Parameters *V_s_* and *K_s_* are the state specific kernel width and shape, respectively, as further discussed below. The model assumes that the probability of an origin county is proportional to the number of premises within the county and the probability of a destination depends on distance from origin county, the number of premises within the destination county and historical inflow of animals to a state, *s*. We therefore define the attraction of county *i* based on past inflow to be, 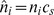, where 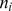 is the number of premises in county *i* (located in state *s*) and *c_s_* is the mean number of animals from the historical inflow into state, *s*, per premises. The historical inflow is reported as total number of cattle and to obtain production type estimates, we assume that this is divided between dairy and beef in proportional to the number of premises of each type in the state.

We assume no biases in observing intrastate vs. interstate movements and the probability of a movement from county *ω* to county *δ* in our model is:
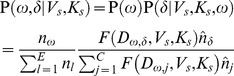
(2)where 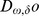 is the distance between *ω* and *δ* based on county centroids, 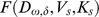, is the distance-dependent kernel model, *E* is the number of counties in state, *s*, and *C* is the total number of counties in the contiguous U.S. (excluding the origin county), i.e. 3108. Movements may also occur within the same county. This has no effect on the network structure because it does not produce a link between the nodes (counties). For epidemiological modeling it may however be of interest and is included in the model, yet it requires some special treatment and 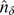 is instead defined as 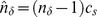 (i.e. we are adjusting 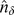 to remove the possibility of a movement having the same destination and origin premises within the county) and 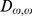 is defined as the mean distance between randomly distributed points in a square of the area of county *ω*, which is approximately 0.52 times the square root of the area.

To quantify the width and shape of the spatial kernel, we use two-dimensional measures of variance and kurtosis, respectively, as defined by [14], [15]. We use a power exponential function to describe the kernel as 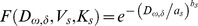 where parameters *a_s_* and *b_s_* are given from *V_s_* and *K_s_* through [15],
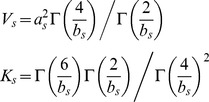
(3)in a continuous, two-dimensional system, the distribution is normalized by 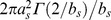. Here, we normalize by summation over all possible origin and destination counties as given by the denominators in equation (2). In this implementation, *K_s_* is of less direct importance for both network properties [16] and predictions for the rate of disease spread [17]. Yet we need to include a kernel with a flexible shape due to possible interactions in the estimation of the width. In this study, we are less interested in the actual parameter values of *V_s_* and *K_s_*, but expressing the model on these dimensions (rather than *a_s_* and *b_s_*) facilitates prior elicitation.

This distribution has some benefits in that it may take the form of some well known distributions as special cases, such as the normal distribution (*b*_s_ = 2) negative exponential (*b*_s_ = 1) and uniform (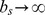). Further, unlike some other commonly used distributions such as the gamma or Weibull distribution, the power exponential distribution (also sometimes denoted as the generalized normal distribution) does not approach either infinity or zero as the distance approaches zero. The lower limit for kurtosis is 4/3, which is the uniform distribution, and we also define 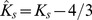.

Through 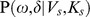 we may assess the conditional probability of 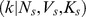 as
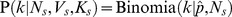
(4)for 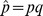, where *q* is the proportion of interstate movements analyzed ( = 0.1 since we observed 10% of the interstate movements) and
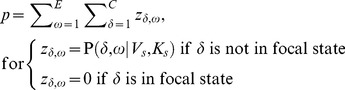
(5)i.e. we are summing up all the interstate probabilities. Further, modeling of observed intrastate movements from state s is given by



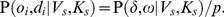
(6)In formulating a Bayesian model, we implement hierarchical Bayesian modeling of ***V*** and ***K***. This implementation improves the parameter estimates for states with few movements by “borrowing strength” [18] of kernel parameters from other states. The full Bayesian model is written as
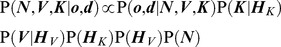
(7)

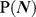
 is the prior of N and 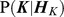 and 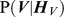 are hierarchical priors with hyper parameters ***H***_K_ and ***H***_V_, respectively with hyper priors 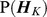
and 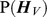, respectively. Here we use ***H***_V_ and ***H***_K_ to generally refer to the hierarchical prior parameters in the model. In the next section we elaborate on the choice of priors. Table 1 presents an overview of the main parameters of the model.

**Table 1:** Model parameters.

	Description	Source for estimation and comments
Estimated state level parameters		
*V_s_*, *K_s_*, *N_s_*	State (*s*) specific width (*V_s_*) and shape (*K_s_*) of spatial kernel and total number of shipments (*N_s_*).	Estimated jointly, conditional on all data as well as hierarchical parameters for *V_s_* and *K_s_* and a fixed prior for *N_s_* (see text). ***V***, ***K*** and ***N*** denotes parameters for all states.
Hierarchical parameters		
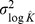 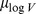 _, 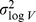 , 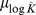 ,_	Mean ( 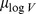 , 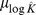 ) and variance ( 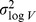 , 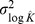 ) for prior distributions of ***V*** and ***K***.	Estimated in the analysis and allows for borrowing strength between state level parameters of *V_s_* and *K_s_*. Conditional on ***V*** and ***K*** as well as hyper priors (see text).
Fixed parameters		
*c_s_*	Mean number of animals/year received from interstateinto state s.	Given by NASS data.
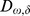	Distance between counties 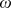 and 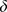 .	Given by NASS data.
*n_i_*	Number of farm in county *i*.	Given by NASS data.
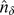	Inflow attraction of county *i*	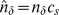 _Calculated as_

### 2.2.2 Elicitation of Priors

In a Bayesian framework, we usually know something about the system, and we incorporate this knowledge to construct a vague prior. Because we implement a hierarchical Bayesian model for the kernel parameters, we do not need to specify priors for parameters of the different states separately. However, we need to specify the hyperpriors.

We define the hierarchical prior for kurtosis 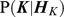 as a normal distribution on the log scale of 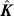, with parameters mean 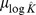 and variance 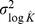. When electing the hyperprior for 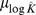, we first note that animal movement in the U.S. consists of both local movements as well as long distance movements across the country. Secondly, we note that animal movements in other countries are typically highly leptokurtic [19] [12]–[13]. Hence, we argue that there should be a low probability for generally platykurtic distributions, i.e., 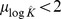 (the two-dimensional Gaussian distribution has a kurtosis of two). Although we expect a heavy tailed distribution, we further argue that the average kurtosis is unlikely to be higher than 100 (as a comparison, the exponential distribution has a kurtosis of 3.33). We want to include some probability of values outside this range and specify the hyperprior 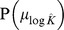 as a normal distribution with approximately 95% of the probability density within this range. Because we are describing the prior on the log scale of 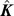, 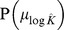 is defined by its mean, 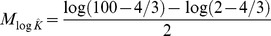, and variance 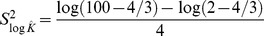 (i.e., approximately 95% of the central probability density of a normal distribution is found within two standard deviations on either side of the mean.).

The conjugate prior for the variance of the normal distribution is the scaled inverse chi square distribution. When specifying the hyperprior of 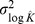 we implement a routine suggested by [18] where the parameters are given implicitly from our prior beliefs about the most likely value (i.e. the mode, 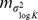) and some upper value, 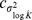, below which we believe that 95% of the probability density is located. To decide on our beliefs about the mode, we start by addressing the range in which we expect to find 95% of the kurtosis estimates of individual states. We argue that one order of magnitude either way is reasonable. Hence, if 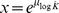, we expect to find 95% of 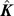 within the range 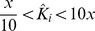. We are expressing this hyperprior on the log scale of 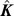 and specify the mode of the hyperprior as 
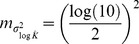
, again from the notion that 95% of the central probability density lies within two standard deviations on either side of the mean. We however want to be vague about this prior belief and specify the upper limit 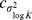 by two orders of magnitude, i.e. 
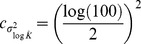
.

We express the hierarchical prior 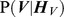 as a normal distribution on the log scale of ***V***. Hence we have two hyperparameters; mean (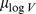) and variance (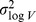). We want a generally vague prior and specify both 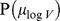 and 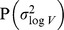 as being proportional to one.

The prior for ***N***, is chosen to be 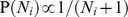, where we use 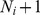 because we include the possibility of zero movements. This gives a lower bound for a large value. However, we give equal probabilities in terms of order of magnitude. For example, the prior probability of there being between 101 and 1000 movements from one state is approximately the same as there being between 1001 and 10000. While this prior becomes somewhat unrealistic for both very low and high values of *N_i_* we argue that it is suitable as a vague prior on the support of the parameters.

### 2.2.3 Markov Chain Monte Carlo Estimation

We analyzed beef and dairy movements separately using the above framework. We separated the two due to the potentially different movement drivers underlying the two production types. Technically, the Bayesian analyses were performed with MCMC, using Metropolis-Hastings updates for ***N***, ***V*** and ***K*** and Gibbs sampling for hyper parameters. We implemented joint updates of each pair 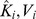 with Gaussian random walk proposals on the log scale of the parameters (conveniently the same scale as the priors are expressed on and we may disregard the determinant of the transformation in the acceptance ratio). Because 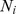 is discrete, we proposed candidate values from a Poisson distribution with mean given by the current position. This is a non-symmetrical distribution and we adjusted the acceptance ratio accordingly.

For each production type (beef and dairy), we ran ten replicates of the MCMC simulation, each with 250000 iterations. For each simulation, the first 50000 iterations were discarded, and the chains were analyzed to ensure that they converged to the same area of high posterior density. Our posterior was given by combining the result of the ten chains. Inference based on MCMC involves repeatedly drawing random numbers from the posterior distribution. These are then used to parameterize the model when generating networks. For further details on MCMC, see [20].

### 2.3 Posterior Predictive Distribution and Network Analysis

There are several ways to validate models in a Bayesian framework. Here, we employ a commonly used method where the observed data and posterior predictive distribution are compared by appropriate summary statistics [18]. Because our aim is to scale up a partially observed network, we used relevant network statistics for comparison between observed and predicted networks as well as randomized networks (described in 2.4). We therefore generated 1000 network replicates by parameterizing equation (7) by random draws from the posterior distribution. Technically this is done by a joint draw from the MCMC output. In order to obtain comparable networks we took 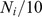 random draws of interstate movements from each state. Our main interest lies in comparison of the whole network structure and we therefore combine the dairy and beef networks. We compared seven network metrics: in degree, out degree, betweenness, diameter, reciprocity, transitivity, and degree assortativity. At the node level, in degree is the total number of shipments that a county, *i,* receives; out degree is the total number of shipments that a county, *i*, sends; and betweenness is the number of shortest paths between all pairs of connected counties that pass through a county, *i*. At the network level, diameter is the maximum number of edges taken to reach any two nodes by the shortest path, reciprocity is the proportion of edges for which there is another edge in the opposite direction (i.e., node *i* to *j* and node *j* to *i*), transitivity is the probability that any two neighbors of a node (i.e., connected by an edge) are connected themselves (also known as the clustering coefficient), and degree assortativity is the correlation of the total degree (in+out degree) of the nodes at the ends of every edge.

Because the validation necessarily compares samples of interstate county-county links (observed and generated) we cannot make comparisons about the presence or weight of individual county links. However, we can make direct comparison between links aggregated to the state-to-state level to evaluate the precision of our model at a large geographic scale. In addition, the summary of cattle movements at the state scale has been previously reported [21]. We determined the similarity of the number of directed links between states by using a mantel matrix-correlation test between the observed ICVI state-to-state adjacency matrix and each of 1000 *N_i_*/10 samples of generated networks and 1000 *N_i_*/10 samples of randomized networks (see 2.4). We determined significance of the correlation (null hypothesis, *r* = 0) with 999 random permutations of the observed ICVI adjacency matrix.

### 2.4 Randomized Network Construction and Comparison

In order to compare observed and kernel model generated data to an appropriate null, we also generated randomized networks for comparison. For each state we generated the same number of outgoing movements as the number of observed movements (as given by the ICVI data) for that state. For each movement, the origin county was picked randomly within the state and the destination was picked randomly from all other counties.

## Results

### 3.1 Posterior Distributions

Our main interest does not lie in the parameter estimates themselves, but rather in how well the method performs in predicting the network structure. Hence, we focus on a general description of the estimates, and marginal posteriors of parameters are presented in the supplementary material. The estimated movement kernels were generally leptokurtic with 93.9% of the estimated marginal densities of kurtosis higher than two (i.e. the kurtosis of a normal distribution) and 87.3% larger than 3.33 (i.e. the kurtosis of an exponential distribution). The result however revealed very diverse kurtosis estimates. For dairy movements, the lowest median kurtosis was estimated for Massachusetts at 1.42 [1.33, 34.1] (number in brackets indicate 95% central credibility interval of estimated kurtosis) and the highest for Texas at 1.28×10^5^ [4.52×10^3^, 1.65×10^6^]. The corresponding values for beef movements were found for Mississippi with 1.41 [1.39, 1.46] and Iowa with 7.03×10^6^ [2.14×10^6^, 6.77×10^8^] (Figure S1).

The lowest kernel variance for dairy movements was estimated for Massachusetts with median 5.81×10^4^ [4.06×10^4^, 4.69×10^5^] km^2^ and the highest for Texas with 1.64×10^9^ [1.07 ×10^8^, 3.90×10^10^] km^2^. The corresponding values for beef movements were found for Connecticut with 1.13×10^4^ [2.54×10^3^, 4.50×10^5^] km^2^ and Kansas with 2.40×10^10^ [1.54×10^9^, 1.88×10^11^] km^2^ (Figure S2).

While the main focus of this study is not to compare the dairy and beef industry, modeling the production types separately illustrated heterogeneity in the shipment characteristics among beef and dairy production. Using 95% probability as a level where we consider having strong support for differences, five states (Connecticut, Michigan, Minnesota, New Mexico and New York) showed strong support that more dairy than beef movements originated in that state, while 32 states showed strong support that more beef than dairy movements originated in that state (Figure S3). In terms of width and shape of the kernels, ten and four states showed strong support for larger *V_i_* and *K_i_*, respectively, for dairy movements whereas 12 and 14 states showed strong support for larger *V_i_* and *K_i_*, respectively, for beef movements (Figure S1, S2).

The results for the total number of movements per state, *N*, are more transparently presented by the ratio *N/*(10*k*), i.e. the ratio between the total number of predicted intra-state movements and the observed interstate movements multiplied by ten (because we only observe 10% of interstate). Hence, a high value is interpreted as a state having a large proportion of total movements stay within the state. The lowest value for dairy movements was estimated for Rhode Island at median 0.90 [0.26, 2.37] and the highest for Minnesota 7.24 [5.73, 9.15]. The corresponding values for beef movements were estimated for Mississippi with 1.00 [0.87, 1.12] and Texas with 5.93[5.17, 6.77] (Figure S4).

### 3.2 Model Validation

#### 3.2.1 Validation at network level

**Figure 2:**
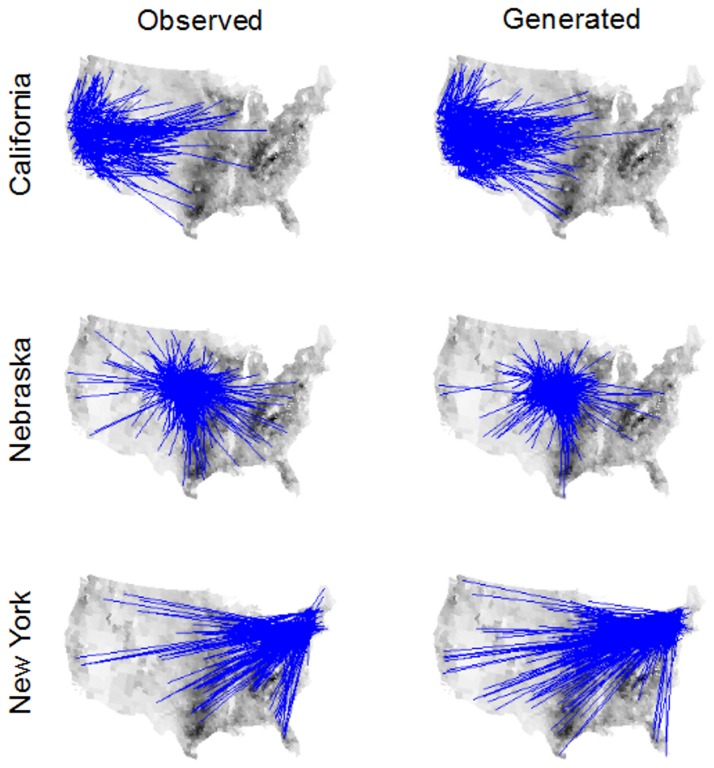
Visual comparison for three example states shows that cattle movement networks generated from the Bayesian distance kernel model (right panels) and cattle movements observed from 2009 Interstate Certificates of Veterinary Inspection (ICVI; left panels) are similar. The observed movements are from a systematic 10% sample of ICVIs from each state and the generated movements are 10% of interstate movements sampled from a single realization out of 1000 kernel generated networks. Darker shading represents the number of cattle premises per county.

**Figure 3:**
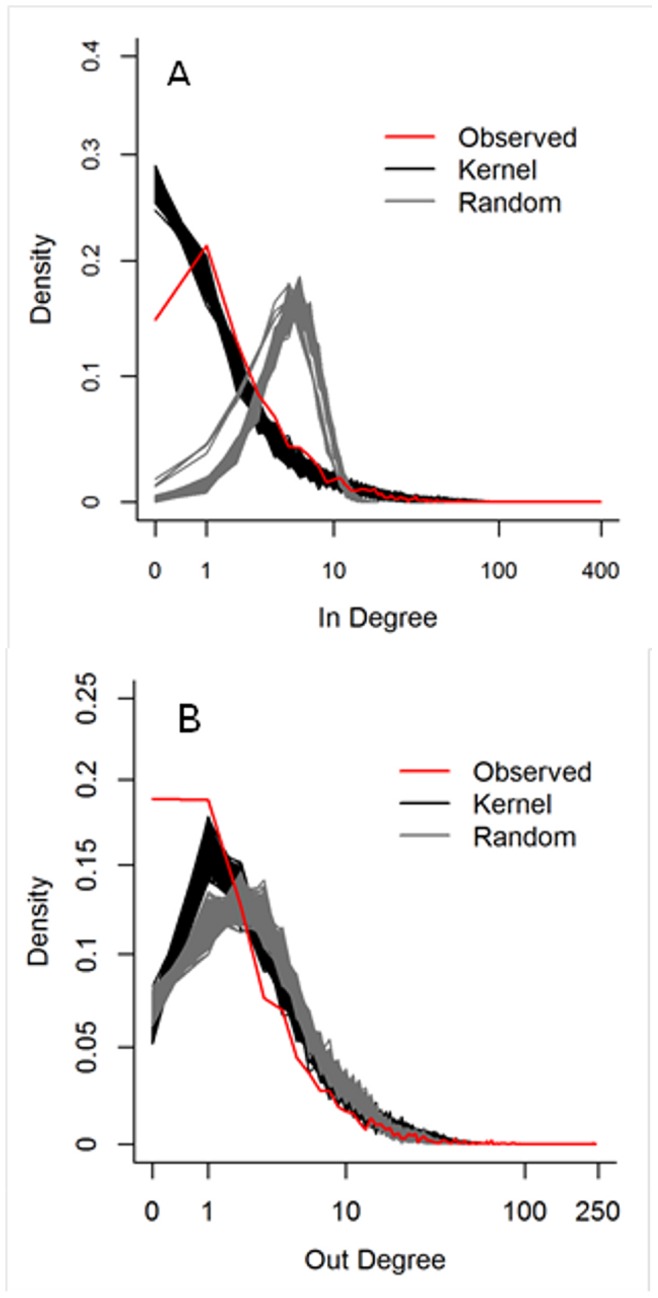
Comparison of the in-degree (A) and out-degree (B) distributions of the cattle shipment networks generated from the 1000 realizations of the Bayesian distance kernel model (black lines), 1000 realizations of randomized networks (gray lines), and cattle shipments observed from 2009 Interstate Certificate of Veterinary Inspection records (red line).

**Figure 4:**
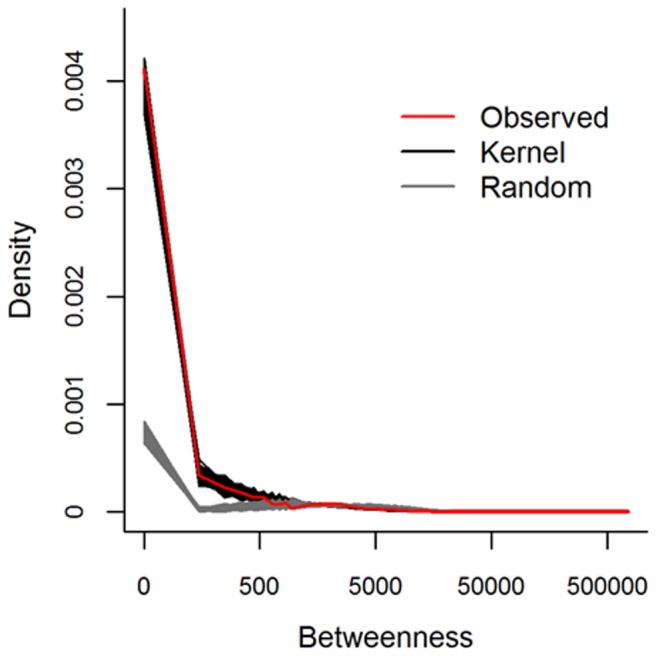
Comparison of the betweenness centrality scores of the cattle shipment networks generated from the 1000 realizations of the Bayesian distance kernel model (black lines), 1000 realizations of randomized networks (gray lines), and cattle shipments observed from 2009 Interstate Certificate of Veterinary Inspection records (red line). The betweenness score is a count of the number of shortest paths between any two nodes in a network (*i,j*), that pass through a node (*k*).

To validate the Bayesian kernel model prediction against the data using network properties we generated a comparable 10% sample of interstate movements from full kernel generated networks (section 2.3). Overall, generated networks from the Bayesian kernel model have network statistics that are similar to the observed data and different from randomized networks (Table 2). The sampling of the kernel generated networks resulted in approximately equal numbers of edges (mean [+/−2 Std. Dev.] = 18596.4 [18326.9, 18865.9]) as found in the observed 10% sample (18590), as well as similar numbers of active nodes (counties) (mean [+/−2 Std. Dev.] = 2718.4 [2692.8, 2744.1] compared to the observed number of active counties, 2407). The method to create randomized networks fixed the number of edges equal to the observed data and generated more active counties compared to the observed and kernel generated networks (Table 2). Hence, the overall size of the observed and kernel generated networks were similar with 13% more active nodes in the kernel generated networks (Table 2).The qualitative performance of the kernel generated networks visually matched the observed interstate edges (Figure 2). Quantitatively, the observed in- and out- degree distribution fell within the generated degree-distributions over much of the range with slight deviation between the observed and generated distributions at the lowest and highest degree values (Figure 3). Our kernel generated in-degree distribution overestimated the probability of nodes with no observed in-edges (Figure 3A; these are necessarily nodes with at least one out-edge) and underestimated the large in-degrees at the tail of the distribution (observed max. In degree = 396, mean generated max in degree [+/−2 Std. Dev.] = 185 [165.5, 204.4]). Conversely, the kernel generated distributions underestimated the probability of nodes with no observed out-edges (Figure 3B; these are necessarily nodes with at least one in-edge) and also underestimated the large out-degrees (observed max. Out degree = 242, mean generated max out degree [+/−2 Std. Dev.] = 75.7 [62.8, 88.6]). The observed distribution of betweenness also matched the generated betweenness distribution over most of the range, with some underestimation of the upper tail (observed maximum betweenness = 673608, mean generated max. betweenness = 320256 [152401, 488112], Figure 4). Kernel generated and observed networks had very low transitivity and reciprocity (Table 2). Finally, the mean diameter of the kernel generated networks was 38% larger compared to the observed diameter, although the observed diameter was only slightly below (0.3) the lower bound of the 95% credible interval of the kernel generated network (Table 2).

**Table 2:** Observed global properties and summary node statistics of the Interstate Certificate of Veterinary Inspection network compared to the mean of a 10% sample of inter-state movements from 1000 kernel generated networks and 1000 randomizations of the observed data.

Statistic	Observed value	Kernel Mean	Standard deviation	Randomized Mean	Standard deviation
Number of active nodes (counties)	2407	2718.44	12.8	3108	0.655
Diameter	12	16.56	1.79	11.22	0.704
Reciprocity	0.029	0.028	0.001	0.001	0.0002
Transitivity	0.049	0.035	0.001	0.005	0.0002
Mean In/Out Degree	7.72	6.84	0.06	6.19	0.001
Max In Degree	396	184.97	9.73	16.79	1.20
Max Out Degree	242	75.67	6.46	40.88	2.66
Mean Betweenness	5539	6185	226	10914	75.9
Max Betweenness	673608	320257	83928	98022	12406
Assortativity	0.204	0.190	0.016	−0.294	0.016

The kernel generated networks generally performed better than their randomized counterparts. The in-degree and betweenness distributions (Figures 3A and 4, respectively) of the kernel estimates matched the observed distribution much better than the randomized networks, and the match of the out-degree distribution was marginally better (Figure 3B). All but one of our kernel derived network statistics were closer to the observed estimates (Table 2), with diameter as the only exception. The difference in diameter is, however, of small magnitude and is likely due to the randomized networks being based on exactly the same number of movements as the observed, whereas this varies in the kernel generated networks.

#### 3.2.2 Validation at state level

**Figure 5:**
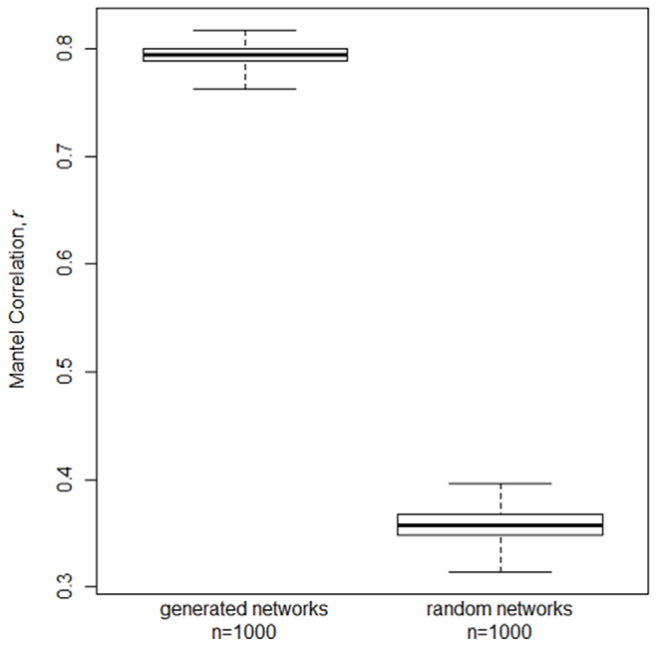
The state level observed Interstate Certificate of Veterinary Inspection data and the *N_i_*/10 sample of interstate movements from 1000 kernel generated networks were highly correlated and consistently more correlated to the observed data than randomized networks. The heavy line in the boxplots represents the median value, the box area represents the 25^th^ and 75^th^ percentile of the data and the whiskers represent the maximum and minimum values.

The kernel generated movements continued to match the ICVI data much better than its randomized counterpart when comparing movements aggregated to the state level. The kernel generated state-to-state level movements had a high correlation with observed data (*r* range: 0.76–0.81) and consistently higher correlation than the randomized networks (*r* range: 0.28–0.31, Figure 5).

## Discussion

Modeling processes that are influenced by livestock movement, such as disease spread, requires confident estimates of how animal shipment patterns connect the players in the system. Undersampling and incompletely observed data are common problems facing data-driven efforts, even in the most well-characterized systems, such as the United Kingdom [22]. Here, we presented a Bayesian method that recreated the observed data (10% sample of ICVIs) within a reasonable amount of uncertainty. The method estimates the probability of movements and is a tool both to scale-up a partial dataset of network connections and to fill in regions where no data are available. In this specific case we have used the method to predict movements at the county level, addressing both the lack of within state movements in the data and that only 10% of between state movements were sampled. Filling in these two types of data gaps for cattle movements in the United States are the foundation for generating a U.S. national cattle movement network. This generated network is novel in the method used to create it and it is the first cattle movement network over such a large region as the U.S., with nodes as specific as individual counties. The method models individual movements, hence we considered the data at a fine granularity, and performance of the method was evaluated at a coarser granularity by analyzing network properties (Figure 1).

### 4.1 Kernel Estimation of Inter-state Movements

Our sample of 10% of cattle shipments that crossed state lines represents the best characterization of cattle movement across the diverse industry and geographic extent of the U.S. cattle industry to date. In order to scale up to the complete network, we developed a Bayesian kernel model based on some simple assumptions about the underlying process and fitted the model to this incomplete data. The model was structured so that the kernel parameters (width, *V_s_*, and shape, *K_s_*) varied for each state, *s*, as well as for beef and dairy shipments. The fitted parameters varied over states and production type (see supplement for estimates of individual states), illustrating the importance of specifying flexible state specific kernels that could model movements in both major production types (i.e. beef and dairy) and over the geographic extent of the U.S. cattle industry.

The kernel model generated a network of movements that was comparable to the observed data. Notably, the kernel model was fit to characteristics of individual cattle movements and county characteristics and predicted both node-centric and global network properties. Within the Bayesian framework, this also allowed us to evaluate the accuracy and quantify the error in the kernel model’s performance. Node level network centrality distributions were comparable over most of the range of the centrality values (in-degree & out-degree; Figure 3A–3B). The observed degree centrality was highly aggregated with few extremely high values and neither the kernel model nor the randomized networks captured the level of observed aggregation (Table 2). The kernel model’s ability to predict in-degree was superior to randomized networks (Figure 3A) and matched the observed consistently better, but with a smaller magnitude, when predicting out-degree (Figure 3B). We believe the deviation at the extreme centrality values reflect a process of preferential attachment that is not captured in our model and hypothesize that such an underlying process exists for parts of the U.S. cattle network. This may cause a more aggregated distribution of shipment origins and destinations; such that only a few counties attract or send many shipments and most counties send or receive relatively few shipments.

We postulate that we could not capture this process in our model because it is structured by unobserved characteristics that occur at a scale smaller than our nodal unit (county). For example, the kernel model does not include any information about the types of premises in a county and the presence of certain types of cattle premises, such as livestock auctions or feedlots, may predispose a county to attract more incoming edges or generate more outgoing edges than expected based on a count of premises alone. A kernel generated shipment will have a probability of terminating in a county, *i*, at distance, *d*, following the kernel parameter estimation and, because we are using a spatially explicit model, the probability of the kernel model predicted shipment terminating in neighboring counties to *i* (with comparable number of premises) will be very similar. Hence, a county that receives many shipments may have an under estimated in-degree because many nearby counties receive shipments that, in the observed network, are attracted to the single preferred county.

Comparing global properties of the kernel generated networks of interstate movement also produced a similarly close match to the observed network and out-performed randomized networks in most cases. The kernel generated networks had low reciprocity that closely matched the observed value (Table 2). Although the kernel generated networks slightly under-estimated the transitivity, the value is so low that the difference in the number of connected triads from the generated networks would have very little influence on processes such as disease spread [23]. We think that the smaller observed diameter and greater network size (number of counties) may also be a result of the lack of a preferential attachment process, with low degree nodes connecting to each other rather than to highly central nodes. This deviation also highlights the potential importance of a few very important locations in the network. Even though the kernel generated networks matched most of the distribution of observed betweenness centrality values, the observed network has a few much larger extreme values. We hypothesize that the network diameter is increased by not including such high-betweenness nodes from the kernel model; effectively allowing more nodes to develop with intermediate centralities instead of few nodes with very high centralities. Investigating the mechanisms that predict high-centrality at the node level, such as the presence and number of specific premises types, will be key to improving methods that fill in unobserved and under-sampled networks, as well as yield key insights into the economic and agricultural processes that drive the movement of cattle.

The deviation between the kernel generated and observed networks found at low degrees (i.e. counties that send and do not receive or *vice versa*) is unlikely to have much impact if the kernel generated networks are used for disease transmission modeling because these nodes are peripheral to the network. Both the kernel generated and observed networks had neutral to positive degree assortativity, meaning that the high degree centrality nodes are also the high-betweenness nodes [24]. The kernel generated networks captured most of the betweenness centrality distribution well (Figure 3), excluding the few extreme highly central nodes (Table 2). This suggests that the distribution of the most important network characteristics at the node level were maintained by the kernel model.

At coarse spatial scales, geographic patterns generated by the kernel model were more similar to the ICVI sample than those generated by randomization (Figure 5). Approximately 80% of the links, aggregated at the state-to-state level, generated by the kernel method were identical to the observed ICVI links, with this similarity representing a conservative estimate due to differences in sampling interstate movements for weakly connected states. Importantly, the kernel appears to capture the mass of movements primarily to central states (Figure 3), as is expected from the centralized feedlot infrastructure in the U.S. Thus, the spatially explicit kernel model performed well when predicting destinations at a coarse geographical scale.

### 4.2 Uncertainties, Limitations and the Benefits of the Kernel Approach

The aim of the kernel model is to describe a complex process by a set of parameters that captures essential aspects of the observed contact structure. By doing this within a Bayesian framework, we acknowledge the importance of uncertainty in these parameters and include this when predicting from the model. Future contact patterns may then be predicted based on the assumptions of similar underlying processes. However, as with any data-driven modeling, there are several limitations imposed by the data. Foremost, the data represents a one-year snapshot of a large and fluid industry. We are confident in our ability to explain patterns from 2009, but if there are large scale differences in the contact pattern between years, we might do less well in predicting cattle movement in other (future) years. However, we are encouraged because a comparison of the observed 2009 ICVI data to a coarse grain analysis of interstate cattle movement from 2001 showed that the 2009 ICVI network captured similar patterns of coarse nation-wide animal flow [21]. An additional caveat associated with a single snapshot of data is that it averages over within-year variation. A next step in improving this model is to incorporate information about the seasonality of cattle movement patterns and, by using a Bayesian approach, the network reconstruction can be easily improved with additional data.

An additional assumption is that cattle movements are not influenced by state boundaries, such that the total number of movements (hence, including intrastate movements) may be estimated jointly with the width and shape of the kernel parameterized by interstate movements. This is a difficult assumption to evaluate because a comprehensive measure of cattle movements within states is challenging to obtain. We therefore have to consider that this assumption cannot currently be verified. To address this issue in modeling the spread of infectious disease, any disease-spread model should include sensitivity analysis to address the uncertainty in predicted intrastate movements.

While the estimated network statistics are generally similar to the observed, we have highlighted some potentially important deviations and assumptions that can be used to guide future developments of the kernel approach. The most apparent differences relate to the very high aggregation in network centrality, represented by a few very highly connected nodes that the kernel model fails to reproduce. This is likely to be a result of more complex production structures, where premises of some types have particularly high probability of contact. This may be an important feature for more realistic modeling [25] and we suggest that further developments of the model should include additional factors that are correlated with aggregating cattle movements. We believe that this should ideally be done by identifying node characteristics such as the presence of markets and other infrastructure that play key, but unquantified, roles in aggregating the cattle industry. Future versions of the kernel approach should seek to explicitly model movements to and from such premises.

### 4.3 Impacts for Disease Modeling

The ultimate goal in developing a model that can address under-sampled and missing data is to use the model predictions of cattle movement as a basis for disease-spread models. Our technique extends previous approaches to address sampling of network data by taking a unique focus on a characteristic of sampled edges, without having to sample how node characteristics are involved in the network. Previous approaches to evaluate the effect of sampling network data has relied on knowledge of the characteristics of nodes to fill-in missing edges [26] or evaluate bias based on node sampling. Because our model is based on a characteristic of individual edges (distance of transports), our spatially explicit approach avoided issues that arise from biased sampling of nodes [26] and was able to tractably predict edge weights when the missing data was structurally heterogeneous (i.e. using interstate transports to predict intrastate transports). Also, by using a Bayesian approach to predict movements for disease simulations, a range of likely outcomes can be evaluated because the kernel is a probabilistic description of the system. Further, one may include the uncertainty in the parameters which are preserved and also address the possible range of networks that the data infer.

### 4.4 Conclusions

The ultimate goal in developing a model that can address under-sampled and missing data is to use the model predictions of cattle movement as a basis for disease-spread models. Previous techniques have been concerned with under sampling and are therefore conservative with regard to the network structure [26]. Such approach may be suitable for networks without systematic bias in the pattern of missing links or strong spatial component. Yet, for this system, a spatially explicit approach is required. We also argue that the Bayesian approach is particularly suitable for prediction because it is straight forward to incorporate uncertainly in the sampling.

## Supporting Information

Figure S1 **Marginal posterior estimates of** ***K*** **(measuring kernel shape) by state and production type.** Circles indicate median values and errorbars indicate upper and lower bounds of 95% central credibility interval.
(TIFF)

Figure S2 **Marginal posterior estimates of** ***V*** **(measuring kernel width) by state and production type.** Circles indicate median values and errorbars indicate upper and lower bounds of 95% central credibility interval.
(TIFF)

Figure S3 **Marginal posterior estimates of** ***N*** **(total number of movements) by state and production type.** Circles indicate median values and errorbars indicate upper and lower bounds of 95% central credibility interval.
(TIFF)

Figure S4 **Marginal posterior estimates of** ***N*** **(total number of movements) divided by 10** ***k*** **(i.e. number of movements in the 10% sample multiplied by ten) by state and production type.** Circles indicate median values and errorbars indicate upper and lower bounds of 95% central credibility interval.
(TIFF)
