# Supplementary figures and images for: A Bayesian Approach for Modeling Cattle Movements in the United States: Scaling up a Partially Observed Network
Source: PLoS One. 2013 Jan 4;8(1):e53432. doi: 10.1371/journal.pone.0053432 (PMC3537632; doi:10.1371/journal.pone.0053432)

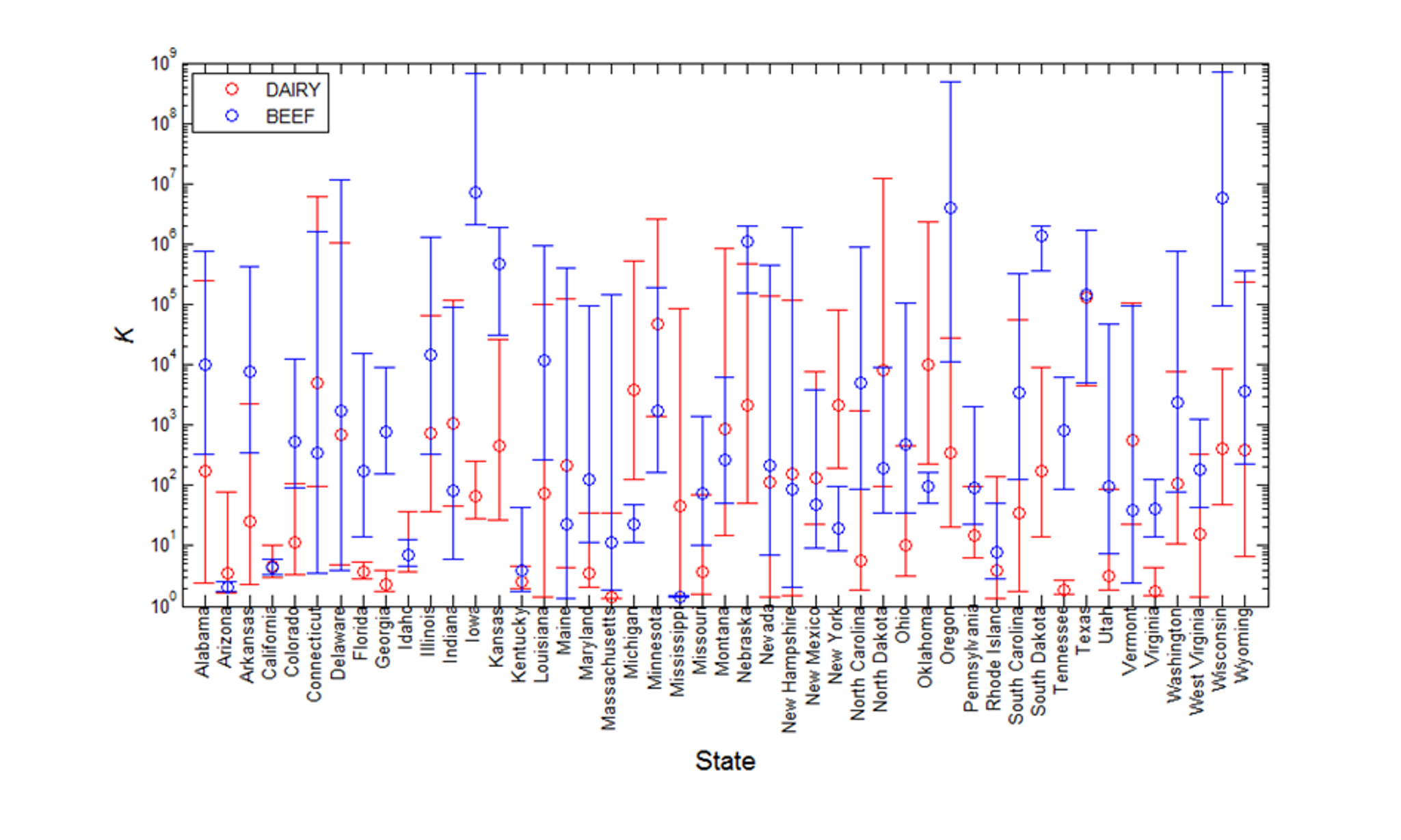

Supplement: Figure S1 — Marginal posterior estimates of K (measuring kernel shape) by state and production type. Circles indicate median values and errorbars indicate upper and lower bounds of 95% central credibility interval. (TIFF) [file pone.0053432.s001.tiff]

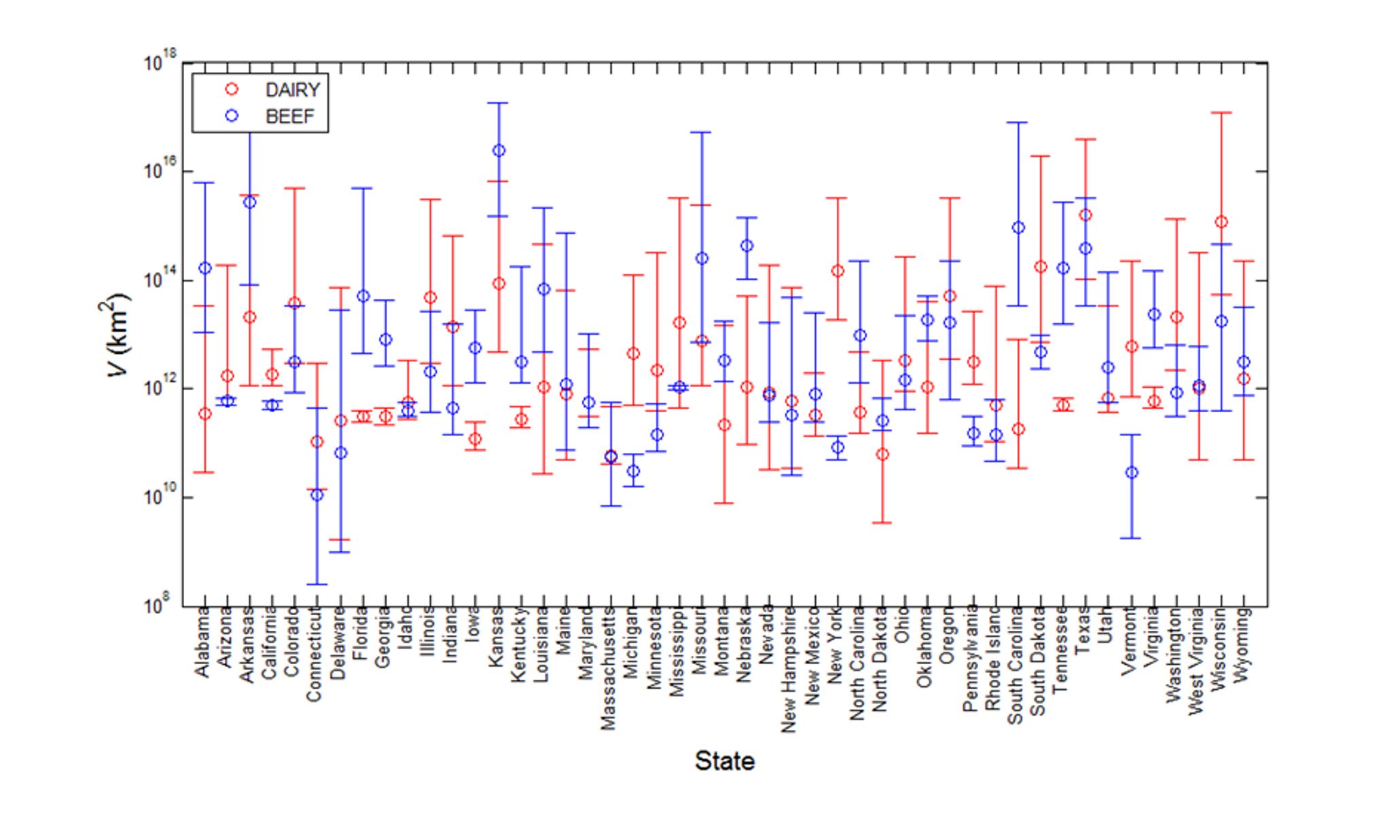

Supplement: Figure S2 — Marginal posterior estimates of V (measuring kernel width) by state and production type. Circles indicate median values and errorbars indicate upper and lower bounds of 95% central credibility interval. (TIFF) [file pone.0053432.s002.tiff]

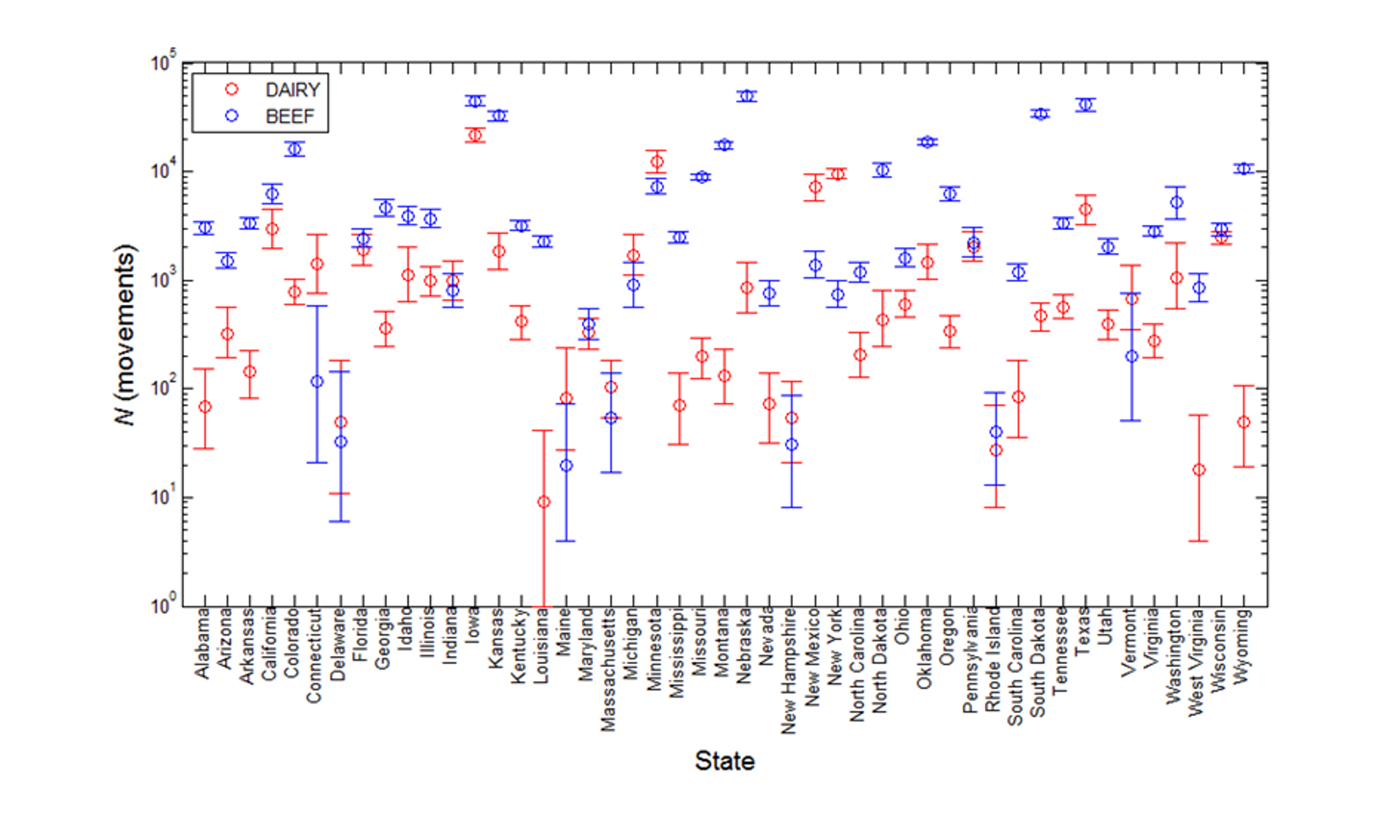

Supplement: Figure S3 — Marginal posterior estimates of N (total number of movements) by state and production type. Circles indicate median values and errorbars indicate upper and lower bounds of 95% central credibility interval. (TIFF) [file pone.0053432.s003.tiff]

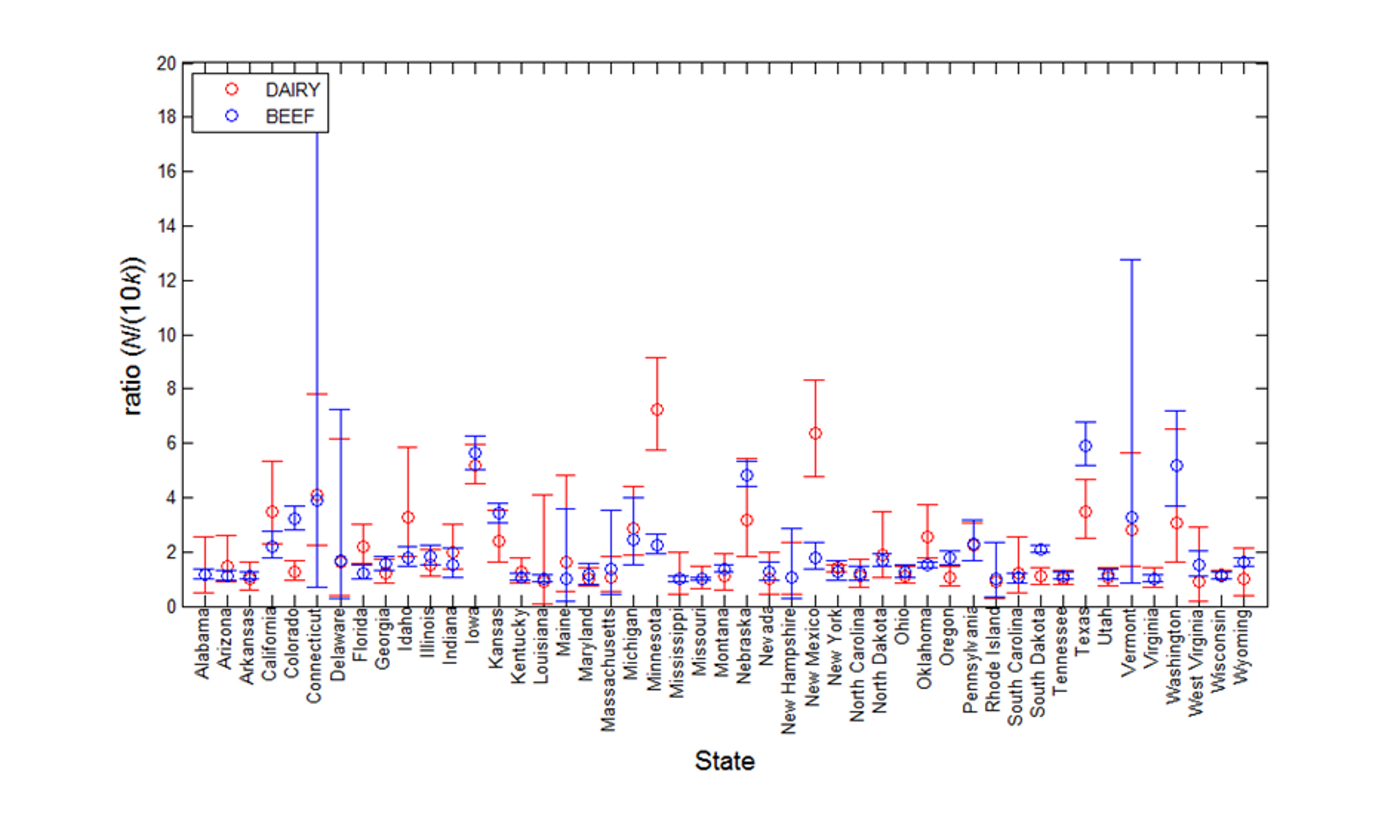

Supplement: Figure S4 — Marginal posterior estimates of N (total number of movements) divided by 10 k (i.e. number of movements in the 10% sample multiplied by ten) by state and production type. Circles indicate median values and errorbars indicate upper and lower bounds of 95% central credibility interval. (TIFF) [file pone.0053432.s004.tiff]
